# Recent Advances in Tissue-Engineered Cardiac Scaffolds—The Progress and Gap in Mimicking Native Myocardium Mechanical Behaviors

**DOI:** 10.3390/jfb14050269

**Published:** 2023-05-12

**Authors:** Somayeh Baghersad, Abinaya Sathish Kumar, Matt J. Kipper, Ketul Popat, Zhijie Wang

**Affiliations:** 1 School of Biomedical Engineering, Colorado State University, Fort Collins, CO 80523, USA; somayeh.baghersad@colostate.edu (S.B.);; 2 Department of Chemical and Biological Engineering, Colorado State University, Fort Collins, CO 80523, USA; 3 School of Materials Science and Engineering, Colorado State University, Fort Collins, CO 80523, USA; 4 Department of Mechanical Engineering, Colorado State University, Fort Collins, CO 80523, USA

**Keywords:** nanofibrous scaffold, composite hydrogel, anisotropy, viscoelasticity, nonlinear elasticity, myocardial regeneration

## Abstract

Heart failure is the leading cause of death in the US and worldwide. Despite modern therapy, challenges remain to rescue the damaged organ that contains cells with a very low proliferation rate after birth. Developments in tissue engineering and regeneration offer new tools to investigate the pathology of cardiac diseases and develop therapeutic strategies for heart failure patients. Tissue -engineered cardiac scaffolds should be designed to provide structural, biochemical, mechanical, and/or electrical properties similar to native myocardium tissues. This review primarily focuses on the mechanical behaviors of cardiac scaffolds and their significance in cardiac research. Specifically, we summarize the recent development of synthetic (including hydrogel) scaffolds that have achieved various types of mechanical behavior—nonlinear elasticity, anisotropy, and viscoelasticity—all of which are characteristic of the myocardium and heart valves. For each type of mechanical behavior, we review the current fabrication methods to enable the biomimetic mechanical behavior, the advantages and limitations of the existing scaffolds, and how the mechanical environment affects biological responses and/or treatment outcomes for cardiac diseases. Lastly, we discuss the remaining challenges in this field and suggestions for future directions to improve our understanding of mechanical control over cardiac function and inspire better regenerative therapies for myocardial restoration.

## 1. Introduction

Heart failure (HF) is the leading cause of morbidity and mortality worldwide and in the US despite many breakthroughs in medicine and biotechnology [1,2,3,4]. Approximately 115 million Americans have hypertension, 100 million have obesity, 118 million have prediabetes or diabetes, and 125 million have atherosclerotic disease, all of which are well-known risk factors for the development of HF. Myocardial infarction (MI), often known as heart attack, is an acute coronary syndrome that results in the formation of non-contracting fibrotic scar tissue and the malfunction or death of cardiomyocytes. The injury is basically non-reversible because of the low regenerative potential of mammalian hearts [1,2]. According to the most recent data from the National Health and Nutrition Examination Survey, an American has an MI approximately every 40 s [3]. Moreover, hypertension, heart valve dysfunction, arrhythmia, and congenital heart diseases are other key contributors to HF. To date, neither pharmaceutical administration nor heart transplantation has been able to sufficiently restore the function of a failing heart. Thus, there is an urgent need to develop new therapeutic strategies, such as tissue regeneration, to rescue damaged cardiac tissues [4,5].

An emerging trend in cardiac regeneration is the use of **cell-based therapy with tissue-engineered bioscaffolds** (e.g., a ‘cardiac patch’) to promote the renewal of myocardial tissues. However, traditional cell or gene therapy requires a single injection of billions of cells or therapeutic molecules, and most of the delivered particles (~90%) are lost in the bloodstream. Even among the homing cells, approximately 90% of the injected cell population dies quickly (e.g., within several hours) even when delivered via intramyocardial injections [4,6]. Tissue-engineered scaffold implantation is advantageous because the scaffold can provide a vehicle for cell colonization, migration, and proliferation, thereby improving cell viability and its therapeutic effects. Scaffolds could also provide mechanical support for the myocardium and enable a controlled release of cells or cell products. An enhancement in other properties of the scaffolds such as electrical conduction, microbiology/biocompatibility, and biodegradation could potentially improve the therapeutic outcomes as well. Moreover, tissue-engineered scaffolds have been used in in vitro studies to elucidate the mechanisms of cellular function, screen for pharmacological effects, and provide a sufficient source of functional tissues before transplantation [4,7,8,9].

The ability of tissue-engineered scaffolds to recreate the native tissue microenvironment, including the topographical, mechanical, electrical, and biochemical characteristics, is important. For example, the heterogenous, complex microstructure in the myocardium is composed of layered and aligned myofibers (cardiac muscles) supported by a dense, highly vascularized extracellular matrix (ECM). At the molecular level, the cardiac ECM supplies biochemical signals via various protein binding sites and the diffusion of secreted paracrine factors. At the cellular level, many cell types sense the local mechanical properties of the ECM through adhesive contacts that are connected to the cytoskeletons. Through such cell–matrix interactions, biochemical signaling pathways inside the cell can be activated, leading to changes in cell shape, cell migration, gene expression, cytokine signaling, and ECM production. These processes, referred to as mechanotransduction, can contribute to stem cell differentiation, tissue remodeling, homeostasis, or disease progression. Mechanotransduction is critically important in cardiac tissue, wherein both the tissue’s passive mechanical properties and the dynamic, active mechanical stresses provide important cues to cardiac cells. At the tissue or organ level, the myocardial ECM provides advanced biophysical features that are linked to the systolic and diastolic function and facilitate the coordinated transmission of electrical signals. Alternatively, cardiac cells grown on rigid (super-physiological or pathological) substrates have rounded (abnormal) shapes, irregular attachments, reduced contractility, and increased apoptosis [10,11]. Thus, a biomimetic scaffold that replicates these properties better promotes cell functions and tissue regeneration [1,10,11]. While the applications of tissue-engineered scaffolds in heart diseases are broad and have diverse purposes, we primarily focused on the mechanical biomimicry of the scaffolds for myocardium regeneration.

Our aspiration in this review was to call for **the development of mechanically biomimetic tissue-engineered scaffolds by implementing the following mechanical features**. First, most studies have utilized isotropic substrates such as hydrogels, which fail to capture the anisotropic elastic behavior of native cardiac tissues. Second, most synthetic biomaterials exhibit only linear elastic behavior, whereas all biological tissues exhibit nonlinear elastic behavior, including the myocardium and heart valve. Finally, despite the growing evidence supporting the role of ECM viscoelasticity in regulating cellular behavior, this mechanical behavior of scaffolds remains largely unexplored in myocardial regenerative research. To advance the development of biomimetic scaffolds, we will discuss how anisotropic, nonlinear elastic, and viscoelastic mechanical characteristics can be achieved by current methodologies. We will also discuss the remaining limitations of the scaffolds, and the current understanding of the impact of these mechanical properties on in vitro cell behavior or in vivo myocardial regeneration.

## 2. Common Methods to Fabricate Scaffolds

### 2.1. Decellularized Tissue Scaffolds

Exploiting nature’s own products is the driving force for using a native tissue ECM architecture repopulated with cardiac and/or vascular cells. Intuitively, an optimal structural and functional environment for cardiomyocytes is the myocardium itself [12,13]. Typically, enzymatic and/or detergent-based methods are used to decellularize a part of or the entire cardiovascular organ to remove the cells while retaining most ECM components. (A detailed review is provided by Gilpin et al. [14]). To date, porcine [15], goat [16], rodent [17,18], and human [19,20,21] hearts have been used to derive myocardial ECM for a variety of research purposes [22]. Among them, myocardial ECM derived from porcine hearts is the most advanced in the translational process, with a product now undergoing clinical trials for post-MI patients (identifier: NCT02305602) [23]. This approach can provide scaffolds with attractive biocompatibility and physiologically relevant structural and mechanical properties. However, this method has limited scalability and inconsistent batch-to-batch quality, preventing its broad use across labs or clinical trials. In this review, we only focused on the fabrication methods mentioned below and their applications in cardiac research.

### 2.2. Hydrogel Scaffolds

In the 1980s, hydrogel materials were pioneered as an advanced culture scaffold for fibroblasts and skeletal muscle cells, later resulting in the first myocardial muscle model system with a collagen matrix by Eschenhagen et al. in 1997 [24]. In addition, fibrin, collagen, laminin, Matrigel, and combinations of various ECM proteins have been used to develop various hydrogel systems for the functional enhancement of engineered tissues, with or without using casting molds and anchoring molecules [8,25]. A key advantage of this method is that the naturally existing ECM components promote cell growth and the development of cell–cell and cell–matrix connections [22,26]. Hydrogels have been widely applied in tissue engineering and regenerative medicine [26,27,28], drug delivery [29,30,31], soft electronics [32,33], and biosensors and actuators [34,35,36]. In general, hydrogels are elastic scaffolds with substantially lower stiffnesses than the native myocardium or heart valves [37]. To overcome the mechanical weakness, composite scaffolds have been developed by blending hydrogels and synthetic biomaterials to develop materials that more closely mimic the mechanical properties of cardiac tissues [38,39,40,41,42].

### 2.3. Electrospun Nanofibrous Scaffolds

Beginning with the Formhals patent, electrospinning has an almost 90-year history and numerous applications in modern industry [43]. Studies on polymer fibers in the 1990s led to the re-recognition of electrospinning and new applications in tissue engineering and drug delivery, mainly due to technological advancements allowing the resolution and moderation of nanometer-scale features [44,45]. Electrospinning is one of the most practical and versatile methods for fabricating micro/nanofibrous polymeric structures with precise control over matrix architectural features, such as fiber size, orientation, crosslinks, and fusion, and the resulting properties, including mechanical and electrical conduction behaviors [46,47,48]. Electrospinning is a widely used mode of nanofiber production because it can be employed to generate nanofibers from a wide variety of both synthetic and biologically derived polymers, polymer blends, and composites [49]. A polymer solution is ejected through a syringe at a specific flow rate onto a metal collector at a desired distance from the needle tip. A voltage is applied between the needle tip and the collector to supply an electric field to draw the polymer fibers [37,50]. The fibrous architecture and properties can be altered by a variety of parameters in the polymer solution (e.g., molecular weight, concentration, mixture of polymers); in the operation of the apparatus (voltage, distance from needle tip to collector plane, injection flow rate, and duration); and in the setup of the collector or other processing conditions (e.g., humidity) [37,51,52].

### 2.4. Three-Dimensional Bioprinted Scaffolds

Three-dimensional printing is the fabrication of three-dimensional objects from digital models by the layer-by-layer deposition of materials onto a surface. It has emerged as a technique for developing 3D scaffolds for tissues or organs with a programmable structure and precise control over the micro/nanostructure and the distribution of tissue components. The capability of 3D printing in micro- and nanoscale fabrications for cardiac tissue engineering was discussed in detail by Kankala et al. [53]. The mixture of cells can be achieved either through a cell seeding procedure followed by the printing of complex scaffolds or the simultaneous delivery of biomaterials and cells to construct 3D cell-laden scaffolds [54,55]. There are three primary ways to achieve 3D bioprinting: inkjet bioprinting, laser-assisted bioprinting, and extrusion bioprinting. The advantages and disadvantages of these methods were reviewed by Xie et al. [56].

## 3. Anisotropic Tissue-Engineered Scaffolds

**Figure 1:**
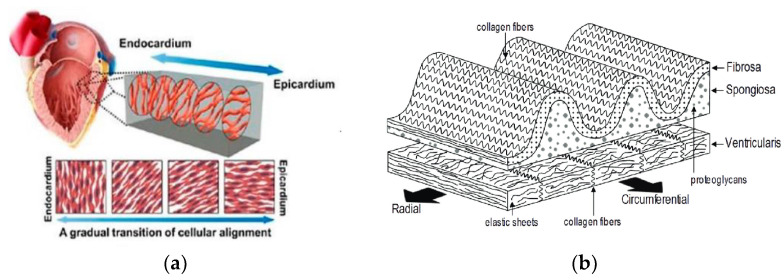
Schematics of (**a**) myocardium showing a gradual transition of aligned cell layers from endocardium to epicardium [38] and (**b**) trilaminar leaflet structure of semilunar valves, illustrating the fibrosa, spongiosa, and ventricularis layers, as well as their principal constituents [63].

Most biological tissues exhibit some degree of anisotropy in their mechanical characteristics. That is, the tissue’s mechanical behavior is different in different directions. This feature results in direction-dependent cellular activities such as cytoskeleton rearrangement and alignment, integrin activation, and ECM deposition. In terms of bulk mechanical behavior, tissue anisotropy varies from almost zero (isotropy) in tissues such as the liver to a high degree of anisotropy in tissues such as ligaments and tendons. Cardiac tissues, including the myocardium and heart valves, are anisotropic as well. The ventricular wall is a multi-layer tissue with complex microstructures in which cardiac muscle fibers are interconnected hierarchically within collagen fibers. The variation of the main fiber angle across the ventricular wall is responsible for the longitudinal and circumferential motion of cardiac torsion (Figure 1a) [12,57]. These characteristics result in mechanical and electrical features that are directionally dependent—a phenomenon known as cardiac anisotropy. The transmural variation in the myofiber/collagen has been confirmed by the examination of serial histology sections from the rodent and ovine myocardium [58,59]. With disease progression (such as hypertension), the fiber alignment is further altered, and the tissue becomes more anisotropic [58]. The fiber organization is essential for the organ’s mechanical and electrical functions, and an alteration may lead to organ dysfunction and eventually HF. Moreover, the structure of heart valves is complex, yet well-organized, with three distinct layers (ventricularis, spongiosa, and fibrosa) that each serve a specific function (Figure 1b). The ventricularis layer, located on the ventricle side, is mostly composed of radially aligned elastin fibers. In the spongiosa—the middle layer of the native valve ECM—randomly aligned proteoglycans are present. The fibrosa layer is dominated by dense collagen fibers with circumferentially oriented structures. As a result, the valve tissues exhibit anisotropic mechanical, biochemical, and biophysical functions [12,57]. Tissue-engineered scaffolds for cardiac regeneration or studies of the biomechanical mechanism of HF must employ a similar microstructural organization. The fabrication methods to produce anisotropic scaffolds for wide applications in tissue engineering have been recently reviewed [60,61,62]. In this paper, we mainly focus on the myocardial applications.

### 3.1. Methodology to Induce Anisotropy in Scaffolds

Mechanical anisotropy in a scaffold can be imparted by fiber alignment and organization. To date, the methods to generate aligned, anisotropic scaffolds can be classified into the following categories: electrospinning with a rotating collector, gap electrospinning, and 3D bioprinting. Brief descriptions of the main strategies and examples of each category are provided below.

#### 3.1.1. Electrospinning Using a Rotating Collector

**Figure 2:**
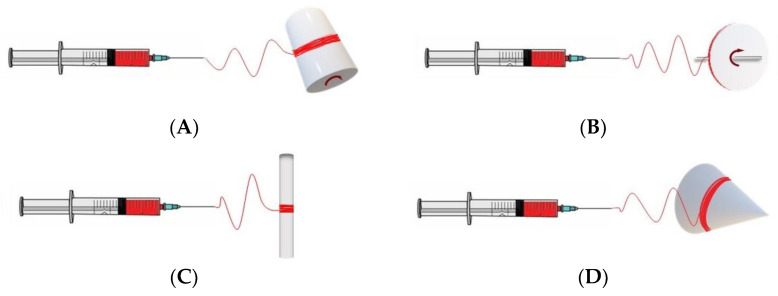
Schematics of modified electrospinning setups using a rotational collector on a (**A**) cylinder mandrel, (**B**) disc collector, (**C**) rotating rod, and (**D**) conical mandrel.

Electrospinning utilizing a rotating collector permits the modulation of fiber alignment through alterations in the geometry and/or rotational speed of the collector. A rotating cylinder mandrel is the most commonly used method (Figure 2A), although it does not provide the highest degree of alignment compared to other methods (Figure 2B–D). In this method, the linear speed at the surface of the rotating drum (i.e., rotating velocity) should match the solvent evaporation rate. The kinematics of the mandrel are determined by the category of processing parameters, which further influence the arrangement of nanofibers (alignment, fiber size, etc.) on the collecting surface [64,65].

**Figure 3:**
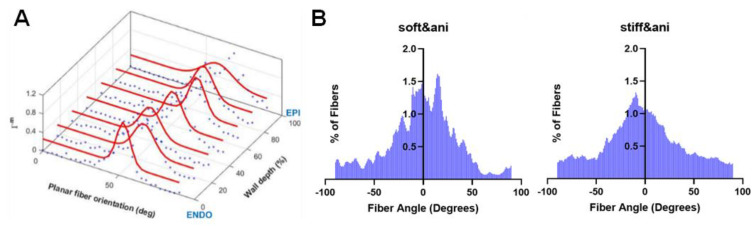
(**A**) A representative figure for the 3D distribution function of myo/collagen fiber orientation in an ovine right ventricle (RV) free wall. The data were obtained from the serial histology section and picrosirius red staining of RV tissue [68]. (**B**) Representative results of nanofibrous alignments in the anisotropic soft and stiff polyester urethane urea (PEUU) scaffolds (collected from rotation mandrel) from SEM images. Data collected in Wang lab.

Achieving fiber alignment requires the careful selection of the processing conditions when using a cylinder rotating mandrel to achieve fiber alignment. First, the induction of fiber alignment occurs within a narrow range of the rotational speed (e.g., between 3.0 and 10.9 m/s) [65]. When the rotating speed is lower than the take-up speed of the fiber, randomly oriented fibers are formed on the drum. When the rotating speed is too high, the depositing fiber jet breaks, and this prevents continuous fibers from being collected [66]. Secondly, within this range, an increasing rotational speed results in more aligned nanofibers. The fiber alignment typically presents a normal distribution of the fiber angles, and the degree of anisotropy is determined by the histogram profile of the fiber angles on the sheet [58,67]. This feature can be viewed as an advantage because the myofibers/collagen fibers from the histological measurement of native myocardium exhibit the same pattern (Figure 3) [68].

To further enhance the fiber alignment, some researchers have utilized a rotating disc (Figure 2B). In this setup, the thin edge of the collector concentrates the electric field, permitting the deposition of highly aligned fibers thereon. The charged jet is restricted within the edge because the electrostatic field between the sharp edge point (+) and needle (−) becomes the strongest in this location. However, highly aligned fibers can only be formed in a relatively small region, and this severely limits the size of the scaffold that can be fabricated [69,70,71]. Like in the cylinder mandrel setup, one should note that the rotating speed not only affects the nanofiber alignment but also the fiber diameter and porosity and, ultimately, the bulk mechanical properties.

Additional modifications to the collector enable the replication of the 3D geometry of the tissue. For example, a 3D tube construct can be formed for vascular graft applications using a small-diameter rotating rod (<5 mm) (Figure 2C) [72,73]. This makes it possible to employ distinct polymers for different layers without the need for further assembly, which replicates native vessel characteristics [73]. Using a conical mandrel (Figure 2D) allows the fabrication of scaffolds with curvilinear microarchitectures that mimic heart valves [74].

#### 3.1.2. Gap Electrospinning

**Figure 4:**
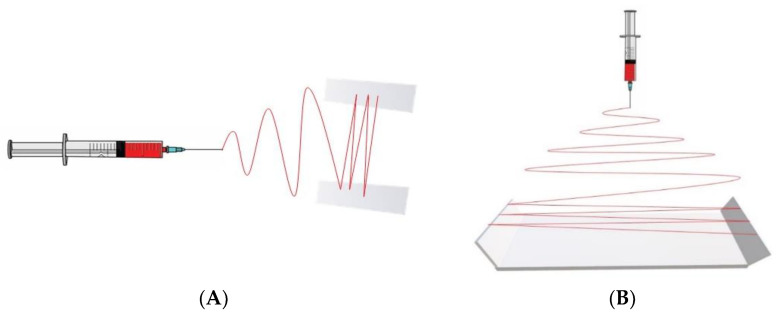
Schematics of fiber alignment achieved by gap electrospinning using (**A**) parallel electrode collectors and (**B**) U-shape electrode collector.

Gap electrospinning induces aligned nanofibers by an applied electrical field. By applying a positive voltage to the polymer solution and a negative voltage to two neighboring plates separated by a gap, the fibers are deposited and stretched from one plate to the other due to the residual electrostatic repulsion between the plates (Figure 4A). Numerous alterations have been made to the basic setup to achieve variations in the microarchitecture, and these were reviewed in depth by Robinson et al. [62]. However, the maximum length of nanofiber sheets has been limited to 10 cm, because large distances inhibit the jet crossing from one side to the other [75,76,77]. To overcome this limitation, Lei et al. recently applied a negative voltage to a U-shape collector and successfully produced long aligned fibers (up to 60 cm) (Figure 4B) [78,79].

Gap electrospinning offers significant benefits in producing controllable, aligned electrospun fibers. It is cost-effective since, in most configurations, no extra equipment is required beyond a typical electrospinning device. In addition, the fiber orientation and gradient of alignment can also be adjusted. However, there are a few drawbacks to the approach. The technology is restricted by the mesh thickness, as the residual charge increases with the mesh thickness. The rise in residual charge causes electrical repulsion and, consequently, a loss of fiber alignment [73]. Finally, since the highly aligned scaffold generally possesses low mechanical strength in the cross-fiber direction, the handling of the thin scaffold is challenging during the removal of the scaffold from the mandrel.

#### 3.1.3. Three-Dimensional Printing

**Figure 5:**
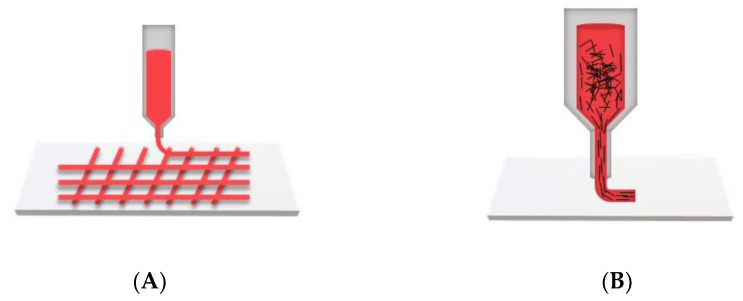
Schematics of 3D printing techniques for fiber alignment: (**A**) direct depositing of aligned and complex architecture scaffolds; (**B**) shear-induced alignment in the scaffold.

Three-Dimensional printing can also be used to induce fiber alignment in anisotropic scaffolds. There are two strategies to deposit aligned fibers: (i) direct depositing into a customized pattern to achieve the complex alignment of micro/nanofibers (Figure 5A) [80,81]; and (ii) the shear-induced alignment of threadlike nanofibers or the elongated deformation of injected components along the printing direction (Figure 5B) [82,83]. Cu et al. [84] printed a variety of designs featuring different fiber widths (100, 200, 400 μm); filling densities (20, 40, 60%); fiber angles (30°, 45°, 60°); and stacking layers (2, 4, 8 layers) to create anisotropic scaffolds compatible with cardiomyocytes. They claimed that the scaffolds accurately represented the transmural fiber alignment and curvature of murine left ventricles.

The advantages of this method include the simultaneous control over the micro-geometry and macro-architecture (such as fiber alignment), the feasibility of achieving a high resolution (~5–50 μm) in the fiber organization, and the proper cell density within the scaffolds [85]. It is important to note that hydrogels are often used and deposited as bioinks to enhance the bioactivity of the scaffold; recent 3D bioprinted cardiac scaffolds were reviewed by Wang et al. (see Table 1 in [86]).

### 3.2. Advantages and Limitations of Current Anisotropic Scaffolds

The incorporation of anisotropy in tissue-engineered scaffolds not only replicates the structural features of native cardiac tissues but also allows for mechanistic studies that can improve our understanding of heart diseases. One important consideration in replicating tissue anisotropy is the fiber angle distribution. As described above, the ventricular wall exhibits a normal distribution of myofiber angles in the tissue sections, and this feature can be achieved by electrospinning with a cylindrical rotating mandrel [12]. In contrast, other approaches including 3D bioprinting generate uniformly aligned or grid structures of fibers that are absent in native tissues. The exact cause and consequences of the normal distribution of myofibers in a single section are not yet fully understood, but a biomimetic cardiac scaffold should consider this feature during scaffold fabrication. Moreover, multiple layers of sheets with varied main fiber angles can be produced either by electrospinning or by 3D bioprinting methods, replicating the myocardium or heart valves with layered, anisotropic characteristics. However, in native tissues, there is also a functional integration of aligned constituents across layers. The current engineering techniques have not been able to provide such in vivo bonding features between aligned layers [87].

A successful biomimetic scaffold should exhibit not only a similar elasticity but also a similar degree of anisotropy to the native tissue. We summarize the reported anisotropy of native cardiac tissues and biomimetic scaffolds in Table 1 and Table 2, respectively. Because of the nonlinear elastic behavior of native tissues, we mainly adopted the tissue elastic modulus measured at low strains that replicate the stiffness of myofibers (in the myocardium) or non-collagen components (in valves). The healthy adult myocardium has an anisotropy degree of 0.3–0.9 in the RV and 0.5–1.9 in the LV, and the fetus myocardium exhibits a higher degree of anisotropy on both sides of the ventricles (Table 1). In addition, the tissue anisotropy is enhanced or even changed (from stiffer in one direction to stiffer in the other direction) with disease progression (e.g., an anisotropy degree of 3.2–5.4 in failing RVs, Table 1). In contrast, tissue-engineered scaffolds have a wide range of anisotropy degrees, ranging from ~2 in a PEUU scaffold to ~46 in a PCL scaffold (Table 2). Except for one study, all the scaffolds presented a high degree of anisotropy (>3) that is absent in the healthy myocardium or heart valves. Thus, there is a lack of consensus on the degree of anisotropy for myocardium tissue constructs, for either healthy or diseased conditions. Another limitation of anisotropic scaffolds is that their Young’s moduli (presented in MPa) are greater than the native myocardium’s Young’s moduli (presented in kPa). Lastly, inducing fiber alignment while keeping other parameters identical increases the bulk stiffness of the scaffold, and thus anisotropic scaffolds are often stiffer than isotropic ones. Therefore, it is important to keep both the elasticity and anisotropy compatible with those of the host tissues.

**Table 1:** Anisotropic mechanical properties of native cardiac tissues reported in the literature. The degree of anisotropy was calculated as the ratio of the Young’s modulus (E) or peak stress between longitudinal (L; main fiber/outflow tract) and circumferential (C; cross-fiber/perpendicular to outflow tract) directions. To distinguish the different orientation systems, the orientation system with the outflow tract and its perpendicular directions are labeled with L* and C*, respectively. Unless stated elsewhere, all data were obtained from healthy animals. LV: left ventricle; RV: right ventricle.

Tissue	Animal Model	Young’s Modulus (kPa)	Anisotropy Degree	Ref.
RV	Fetal porcine	L (5% strain): 17.42 ± 4.86 C (5% strain): 6.30 ± 4.01	2.8	[88]
	Adult human	L (low strain): 2.2 C (low strain): 1.8	1.7	[89]
	Adult ovine	L*: 32–244 C*: 20–248	0.7–0.9	[90]
	Adult rat	L* (low strain): 7.2 (6.7–18.1) C* (low strain): 11.8 (7.1–16.5)	0.6	[91]
	Adult rat (failing)	L* (low strain): 34.2 (18.1–44.6) C* (low strain): 6.3 (5.4–8.6)	5.4	[91]
	Adult ovine	L* (low strain): 7.8 C* (low strain): 25	0.3	[92]
	Adult ovine (failing)	L* (low strain): 86 C* (low strain): 27	3.2	[92]
LV	Fetal porcine	L (5% strain): 16.08 ± 7.08 C (5% strain): 6.87 ± 2.91	2.3	[88]
	Adult Human	L (low strain): 2.7 C (low strain): 1.2	1.9	[89]
	Adult Ovine	L*: 17–178 C*: 19–197	0.5–0.9	[90]
Aortic valve	Adult porcine	L:116 C:170	0.7	[93]
		Radial tension: 1.11 Circumferential tension: 0.26	0.4	[94]
Aortic valve	Adult human	Radial: 2 × 10^3^ Circumferential: 15 × 10^3^	0.1	[95]

**Table 2:** Anisotropic mechanical properties of aligned tissue-engineered scaffolds reported in the literature. The anisotropy degree was calculated by the ratio of longitudinal (L: main fiber) modulus to circumferential (C: cross-fiber) modulus. * poly(l-lactide-co-caprolactone): PLCL; polycaprolactone: PCL; polyester urethane urea: PEUU; poly(vinyl alcohol): PVA; glycosaminoglycan: GAG.

Scaffold	Young’s Modulus (MPa)	Anisotropy Degree	Ref.
PCL/gelatin	L: 48.9	4.7	[96]
	C: 10.3		
PEUU	L: 0.5–1.4	1.4–1.7	[97]
	C: 0.2–1.0		
PEUU	L: 1.4	4.7	[98]
	C: 0.3		
PCL	L: 27.0	14.0	[96]
	C: 1.9		
PLCL*	L: 5.3	6.6	[99]
	C: 0.8		
Nanofiber yarn/hydrogel	L: 110.0	5.5	[38]
	C: 20.0		
PVA	L: 254.0	2.8	[65]
	C: 90.0		
PCL/gelatin	L: 4.8	4.7	[39]
	C: 1.0		
PCL/gelatin-GAG	L: 2.5	8.2	[39]
	C: 0.3		
PCL	L: 18.3	45.7	[100]
	C: 0.4		
PCL	L: 13.8	17.3	[101]
	C: 0.8		
PCL/chitosan	L: 15.0	3.75	[102]
	C: 4.0		

### 3.3. Role of Substrate Anisotropy in Cardiac Tissue

#### 3.3.1. Organ-Level Impact of Substrate Anisotropy

The benefit of using or implanting an anisotropic scaffold for the whole organ function has been reported previously. Mathematical modeling and in vivo studies have shown that anisotropic scaffolds, compared to isotropic ones, enhanced the functionality of a diseased heart by improving depressed LV pump function and increasing systolic function without compromising the filling (diastolic function) [103,104]. Through mathematical modeling, Sallin et al. [105] further demonstrated the significance of myocardial fiber arrangement in the ventricular wall by promoting effective cardiac pumping. When the heart is modeled as an ellipsoid with myocardial fibers oriented in the circumferential (diseased) vs. longitudinal (normal) direction with a helical fiber organization, the ejection fractions are markedly different (30% vs. 60%) and represent those of failing and normal hearts, respectively. Chang et al. fabricated a 3D dual-ventricle bioscaffold with three layers, each with distinct helical arrangements. They showed that the cardiomyocytes (CMs) exhibited appropriate alignments in this scaffold, and the entire construct achieved the spatiotemporal control of excitation–contraction coupling. Additionally, their observation of an increased ejection fraction in the longitudinally aligned scaffold agreed with the results predicted from Sallin’s model. In this investigation, however, the mechanical behavior of the scaffolds did not match that of the native myocardium. The collagen fibers in the natural myocardium coil tightly at small strain rates and uncoil to become stiffer at high strains. In contrast, this 3D scaffold did not reproduce the nanoscale structure of collagen fibers, resulting in straight, bundled fibers that were linearly elastic throughout the strain range [106,107]. We will discuss this limitation in the next Section 4.

#### 3.3.2. Cell-Level Impact of Substrate Anisotropy

Anisotropic structures of native tissues, resulting from the aligned arrangement of ECM components or cells, play an essential role in carrying out and maximizing their direction-dependent physiological functions. Studies probing the cellular responses to anisotropic mechanical environment have been conducted by comparing the outcomes obtained from isotropic and anisotropic scaffolds. The first response of cells to aligned substrates is to change their shape and orientation. Cardiomyocytes cultured on (isotropic) plastic are oriented randomly. As a result, their contractile force is distributed in all directions. However, when cultured in anisotropic scaffolds, the CMs will adopt the fiber alignment and be properly positioned on the scaffold [108]. The elongated cell alignment in turn influences the contractile force as well as cell–cell and cell–matrix interactions. Aligned CMs are also more mature and exhibit a more physiological behavior than randomly distributed cells. For instance, Wanjare et al. [109] co-seeded human iPSC-derived cardiomyocytes (iCMs) and endothelial cells (iECs) onto electrospun polycaprolactone scaffolds with either a randomly oriented or parallel-aligned microfiber configuration. They showed that, in contrast to randomly oriented scaffolds, the aligned scaffolds led to iCM alignment along the microfiber direction and promoted iCM maturation by increasing the sarcomeric length and gene expression of myosin heavy chain adult isoform (MYH7). The maximal contraction velocity of iCMs on aligned scaffolds was significantly greater (3.8 m/s) than that on randomly oriented scaffolds (2.4 m/s). These outcomes demonstrate that anisotropic scaffolds promote CM maturation and contractility.

Other groups have examined the effect of matrix anisotropy on stem or progenitor cell function to elucidate cell mechanobiology and its regenerative potential for the heart. For instance, the role of matrix anisotropy in mesenchymal stromal cell (MSC) behavior and paracrine functions has been investigated. Matrix anisotropy has been shown to play a role in MSC morphology, differentiation fate, and other paracrine functions [110,111,112,113,114,115,116]. Recently, Nguyen-Truong et al. [97] examined the effect of RV tissue mechanics on the pro-angiogenic paracrine function of MSCs, concentrating on the combined effect of RV-like tissue stiffness and anisotropy. Using random and aligned PEUU electrospun scaffolds with the stiffness of normal RVs, they found that the MSCs cultured on the anisotropic group consistently exhibited a higher pro-angiogenic function than those cultured on the isotropic group, showing a positive influence of anisotropy on MSC paracrine function. However, this impact of anisotropy was lacking in the stiff scaffold groups resembling diseased RVs. These results highlighted the importance of the synergistic effect of matrix stiffness and anisotropy in the regulation of MSC function, which may lead to the mechanical conditions of MSC-based treatments for heart failure. Similarly, Allen et al. [117] investigated mouse embryonic stem cell differentiation toward CM regulated by substrate anisotropy. They showed that the cell alignment exhibited a gradient-based response (nonaligned, semi-aligned, and highly aligned) to substrate anisotropy and that an aligned substrate accelerated CM maturation to generate synchronous beating.

## 4. Nonlinear Elastic Tissue-Engineered Scaffolds

Like many biological tissues, cardiovascular tissues exhibit J-shaped stress–strain behavior. This feature is known as nonlinear elastic behavior. For instance, the right ventricle passive stiffness increases nonlinearly with an increased strain/load because of the recruitment of collagen fibers [91]. Disease progression typically leads to CM hypertrophy and the accumulation of collagen, resulting in a leftward shift of the stress–strain curve and elevated elastic moduli in both low- and high-strain regions [92,118]. However, this feature is absent in most of the biomaterials that exhibit linear elasticity. To overcome this limitation, researchers have used a variety of approaches to tune the mechanical properties of materials.

### 4.1. Methodology to Induce Nonlinear Elastic Behavior in Scaffolds

Inspired by biological tissues, the fabrication of crimped, extendable fibers is the main strategy to impart nonlinear elasticity on a biomaterial. One way to induce crimped fibers is by permanently lengthening the sheet along the main-fiber direction first and then returning the sheet back to the pre-stretched length. Meng et al. applied this method to electrospun scaffolds made with polylactocaprone (PCL), poly(lactic acid) (PLA), and poly(l-lactide-co-caprolactone) (PLCL), and they found that the mixture of the three was effective in the formation of crimped structures [119]. In the aligned PLCL scaffold, the fibrous sheet was stretched repeatedly, resulting in permanent elongation. Then, the entire sheet was positioned into the pre-stretched shape, treated with heated ethanol spray, and cooled down quickly to produce wavy nanofibers. This crimped fibrous structure was confirmed by SEM imaging, and the nonlinear elastic behavior was measured by uniaxial tensile mechanical tests. Interestingly, the same methodology failed to generate the crimped fiber structure in the randomly aligned PLCL scaffolds, and thus the nonlinear elastic behavior was absent in these scaffolds. However, using similar methods, Niu et al. electrospun tubular PLCL scaffolds with randomly aligned, axially aligned, and circumferentially aligned structures [120]. They reported nonlinear elastic behavior in all scaffolds. The nonlinearity of these scaffolds was compared and found to be similar to that of native blood vessels (porcine aorta ventralis).

Another way to produce crimped fibers is by controlled heating and/or chemical treatment, as briefly reviewed by Szczesny et al. [121] and Zhang et al. [122]. However, these methodologies often generate scaffolds with low porosity, which results in limited crimped fibers and poor cell infiltration. To improve these aspects, Szczesny et al. electrospun a dual poly-L-lactide (PLLA)/poly(ethylene oxide) (PEO) solution and heated the sheet between two glass slides, either before or after washing the scaffolds to dissolve PEO fibers, with or without poly(vinyl alcohol) (PVA) treatment to increase fiber bonding [121]. They found that only the wash-and-then-heat group exhibited nonlinear stress–strain behavior, whereas the PVA-treated scaffolds failed to present nonlinear elastic behavior. In addition, increased porosity has been found to promote the formation of crimped fibers. In the same study, the authors showed a potential link between porosity and the fiber crimping of the scaffold. Recently, Zhang et al. prepared nanofibrous PLCL/PEO scaffolds and found that the fiber crimping and nonlinear elastic behavior increased with an increase in mesh porosity [120]. This report was consistent with the previous finding of Szczesny et al.

Finally, certain materials may exhibit nonlinear behavior and can be used to fabricate scaffolds. For example, poly(glycerol dodecanedioate) (PGD) is a shape-memory, biodegradable elastomer that is linearly elastic at room temperature but has nonlinear elasticity at body temperature. Ramaraju et al. showed that the incorporation of the small intestinal submucosa (SIS) into the PGD sheets induced nonlinearity in the scaffolds. The mechanical properties of PGD can be tuned with native SIS by altering the thermal curing conditions used. The reason for the nonlinear elastic behavior is thought to be the void spaces formed during the incorporation of SIS sheets into PGD, but increasing the void spaces also decreases the stiffness of the scaffolds [123].

### 4.2. Role of Substrate Nonlinear Elasticity in Cell Behavior

The nonlinear elasticity of matrices changes cell–matrix interactions by regulating cell adhesion, spreading, and signal transduction. Prior studies have shown that cells grown on fibrous ECM with mechanical nonlinearity perceive the mechanical signal distance to be far greater than those grown on synthetic linear elastic polymeric material [119,124,125]. Meng et al. showed that compared to the human umbilical vein endothelial cells (HUVECs) cultured on linear elastic scaffolds, the HUVECs cultured on nonlinear (aligned and crimped) PLCL scaffolds had a greater density of focal adhesions and a higher expression of focal adhesion proteins. This indicated a stronger cell–matrix interaction, which more effectively transduced mechanical signals. These cells also had an increased spreading area, thereby promoting the formation of an endothelial layer on the vascular scaffold. The cell proliferation rate on the nonlinear elastic scaffold was lower than that on the linear elastic scaffold, but it was attributed to the lower Young’s modulus in the nonlinear elastic scaffold [119]. In a separate study, Zhang et al. showed that a nonlinear elastic scaffold promoted HUVEC adhesion and proliferation despite the reduced stiffness of the scaffold. These cellular responses were attributed to the rough surface, increased porosity, and increased hydrophilicity of the nonlinear elastic scaffold rather than mechanical factors [122]. Liu et al. showed that the nonlinearity of the ECM regulated the organization of hASCs by preparing six gels with different concentrations and critical stresses. Finally, Niu et al. cultured HUVECs on nonlinear elastic tube scaffolds with three different fiber orientations (random, circumferential, and longitudinal alignment) [120]. They did not include linear elastic scaffolds as a control, and thus it remains unknown whether the cell proliferation is altered by nonlinear elastic properties.

Crimped fibrous scaffolds promote cell spreading and adhesion, but the effect on cell proliferation remains unclear. However, the mechanisms for altered cell responses are mostly attributed to the matrix topography (rough surface or porous structure) or surface chemistry (hydrophilicity) of the crimped fibrous scaffolds. Whether the mechanical behavior (nonlinear elasticity) is just a side product of the crimped fibers or directly affects the mechanical transduction of the cells is unknown. The exact role of the nonlinear elastic behavior of the substrate in the mechanical signaling pathway of cells should be investigated in future work.

### 4.3. Limitations of Current Nonlinear Elastic Scaffolds

Above, we summarized the current methods for nonlinear elastic scaffold fabrication and some known cellular responses to crimped fibrous scaffolds. While it is encouraging to see the advancement in this biomimetic mechanical property in tissue-engineered scaffolds, it should be noted that the previously mentioned studies focused on applications in soft tissues such as tendons [121], ligaments [126], and blood vessels [127,128]. The fabrication of biomimetic scaffolds exhibiting cardiac nonlinearity remains a knowledge gap. Additionally, the methods for forming crimped fibers need to be improved, as both success and failure to exhibit nonlinear elasticity have been reported in randomly oriented fibrous scaffolds. For example, Meng et al. showed that micro crimped structure formation was only observed in aligned scaffolds (PLCL, PLA, and PCL) and was absent in random scaffolds [119]. However, randomly aligned tubular PLCL scaffolds fabricated by Niu et al. using a similar technique did present nonlinear elastic behavior [120]. Therefore, other factors, perhaps related to the fiber orientation and bonding, may play a role in the formation of crimped fibers and should be investigated. Third, the mechanical mechanism of the ‘nonlinear elastic response’ of cells is still not fully understood, and most researchers have attributed the altered cell behavior to morphological or chemical properties from the crimped fibrous micro-structure of the scaffold. Moreover, prior in vitro studies have been performed in a static environment wherein the ‘crimped’ fibers may not be loaded and become straight fibers. Future investigations of the cell responses under dynamic loading conditions (e.g., from small strain to large strain) will provide a better understanding of the mechanical mechanism. This may be particularly critical for cardiovascular research, as the tissues are under constant dynamic loads, which is different from other non-cardiovascular tissues.

## 5. Viscoelastic Tissue-Engineered Scaffolds

Another less investigated mechanical behavior of scaffolds is viscoelasticity. A viscoelastic material has elastic behavior that is time-dependent and strain-history-dependent. Viscoelasticity is universally present in biological tissues. Heart valves are viscoelastic [129], and more evidence has recently shown that the ventricular free wall exhibits viscoelastic characteristics as well [90]. Using either uniaxial or biaxial tensile tests, hysteresis loops and/or stress relaxation curves are commonly observed in ventricular tissues [90,127,130]. The cardiac tissue viscoelasticity can be attributed to the complex composition of the tissue, which includes cardiac cells (e.g., cardiomyocytes), ECM molecules (e.g., GAGs and collagen), extracellular fluids, and the interactions between these components.

Unlike the increased awareness of the importance of viscoelasticity in cancer research [131], the viscoelastic property of myocardial tissues or tissue-engineered scaffolds is seldom investigated in cardiac research. Thus, in this section, we extend our review beyond the cardiac field and discuss the methods to induce viscoelasticity in hydrogels and/or synthetic scaffolds and some known impacts of substrate viscoelasticity on cellular behavior, in the context of general biological applications. A review of techniques to characterize native or engineered tissue viscoelasticity is available in [128].

### 5.1. Methodology to Induce Viscoelastic Behavior in Scaffolds

Hydrogels are the most commonly used biomaterials for constructing viscoelastic substrates. Hydrogels can be classified based on the source of the polymers—natural ECM biopolymers (e.g., collagen or fibrin hydrogels); synthetic hydrogels (e.g., polyethylene glycol (PEG) or polyacrylamide (PAM) hydrogels); and naturally derived macromolecular hydrogels (e.g., alginate or chitosan hydrogels). Currently, the main approaches used to modulate the viscoelasticity of hydrogels include: (1) crosslinking polymers; (2) altering the polymer architecture, such as length and branching; (3) tuning the composition; and (4) altering the concentration of the polymer or polymer mixture [132].

Crosslinks in polymeric hydrogels can be physical (e.g., ionic or covalent) and can be static or dynamic. Vining et al. generated various alginate–collagen hydrogels via combined ionic and covalent crosslinking at different densities to tune the matrix viscoelasticity. Across a narrow range of moduli (0.25 kPa, 0.5 kPa, and 2.5 kPa), the equilibrium stress relaxation of the scaffolds was similar to that of the native ECM [133,134]. This parameter was increased significantly (>3000 s) by the addition of covalent crosslinks, which indicated a weakening of the viscoelastic behavior of the scaffold. Because ionic crosslinks are weaker bonds than covalent crosslinks and make it easier to induce frictional energy loss during deformation, the stress relaxation is more pronounced in ionically crosslinked hydrogels. Besides physically crosslinked hydrogels, hydrogels such as hydrazone, oxime, and thioester contain chemically crosslinked hydrogels with dynamic covalent bonds, creating a covalent adaptable network that possesses viscoelasticity. Morgan et al. tuned the mechanical properties of the oxidized alginate hydrogels by mixing with different ratios of dihydrazide (to form hydrazone) and bishydroxlamine (to form oxime) to alter the dynamic covalent crosslinks [135]. In general, the more oxime crosslinks, the stiffer the gel (larger storage modulus). A similar trend was found in the viscosity (loss modulus or relaxation time) of the gels. By changing the composition of crosslinks, the viscoelasticity can also be tuned. Richardson et al. synthesized a range of hydrazone crosslinked polyethylene glycol hydrogels [136]. By adjusting the ratio of alkyl-hydrazone and benzyl-hydrazone crosslinks, the average stress relaxation time of the hydrogels varied from hours (e.g., 4.01 × 10^3^ s) to months (e.g., 2.78 × 10^6^ s). Pauly et al. prepared agarose hydrogels containing proteoglycan mimetic graft copolymers with various polysaccharide side chains (dextran, dextran sulfate, heparin, chondroitin sulfate, and hyaluronan) [137]. Agarose gels have a strain-rate-dependent compressive modulus. When either the highly charged polysaccharide heparin or the neutral polysaccharide dextran is added to the gel, the modulus of the hydrogel is unmodified or reduced; however, when the heparin or dextran additive is included in the form of a proteoglycan-mimetic graft copolymer, the modulus is increased. The gels also exhibit stress relaxation behaviors with multiple time constants for relaxation that can be modulated by the structure and composition of the proteoglycan mimic additives.

While hydrogels are the main type of biomaterials used for viscoelastic studies in the literature, there are a limited number of studies investigating the viscoelastic property of synthetic scaffolds. For instance, the viscoelasticity of PCL scaffolds can be tuned by blending natural or synthetic components at different ratios. Kim et al. attempted to tune the viscoelasticity of PCL scaffolds by adding different concentrations of alginate. They showed that the fluidic viscosity of the scaffold increased by increasing the alginate weight fraction in the composites. The storage modulus (G′) of the blended scaffolds was higher than that of pure PCL scaffolds, and it was increased with an increasing alginate concentration (0.1 Pa to 40 Pa at 0–30 wt % of alginate) [42]. Moreover, Peter et al. reported the preparation of a wide range of viscoelastic polydimethylsiloxane (PDMS) scaffolds, and tuning viscoelasticity was achieved by changing the base:crosslinker ratio of Sylgard 184 and the ratio of Sylgard 184 and Sylgard 527 [40]. Increasing the ratio of Sylgard 184 and Sylgard 527 caused decreases in the storage modulus (G′) and loss modulus (G″) of the scaffolds. The use of synthetical biomaterials can overcome the limitations of most natural-material-based hydrogels, i.e., the achieved viscoelasticity range is relatively small and in a sub-physiological range (i.e., lower elasticity and viscosity than native tissues). Shamsabadi et al. used the microsphere sintering technique to fabricate scaffolds for bone tissue engineering using PCL and bioactive glass (BG) 58S5Z (58S modified with 5wt% zinc) [41]. The viscoelastic behavior of the 0% BG (scaffold with only PCL) and 5% BG samples was determined by performing compressive stress relaxation tests. The storage modulus for both samples increased with the frequency. The loss modulus of the 5% BG sample was higher only for frequencies <0.4 Hz. The smaller loss modulus for the 5% BG at higher loading rates indicated its lower viscosity, and because of this, its storage modulus remained nearly constant in this range. Mondesert et al. fabricated fibrous scaffolds with repetitive honeycomb patterns. The relaxation of the scaffolds was tested in directions D1 and D2 at a 15% strain [138]. The scaffolds exhibited a slight relaxation in both directions, showing that the viscosity of the material did not drastically influence the mechanical behavior. Hence, the viscous behavior of these scaffolds was neglected while analyzing their mechanical properties.

### 5.2. Role of Substrate Viscoelasticity in Cell Behavior

Recent pioneering work has revealed some new findings on the impact of substrate dynamic mechanical behavior (viscoelasticity) on various cellular behaviors, including cell morphology and spreading, migration, proliferation, differentiation, and ECM deposition.

#### 5.2.1. Cell Spreading and Migration

Cell spreading is closely related to cell–matrix interactions, which affect the distribution of cell traction forces and mechanotransduction pathways and maintain the mechanical homeostasis of the cell. To examine how cell spreading is influenced by matrix viscoelasticity, Cameron et al. modulated the viscosity (the loss modulus) of polyacrylamide (PAM) hydrogels while maintaining the same elasticity (storage modulus) to study the spreading effect of hMSCs on these hydrogels [139]. Increasing the loss moduli significantly decreased the length of the focal adhesions (FAs), which affected the spreading of the cells. The smaller size of the FAs in hMSCs on more viscous substrates showed that the FAs were less mature and more transient, indicating that the hMSCs were more motile or actively spreading. An additional study with RGD (Arg-Gly-Asp)-coupled alginate hydrogels showed that viscoelastic hydrogels induced a larger spreading area of human MSC than elastic hydrogels while keeping the initial modulus or ligand density constant [140]. Scaffolds with increased creep better promoted the spreading of MSCs on a 2D culture [141]. Similar findings were observed in the 3D culture of MSCs. Enhanced creep led to the increased spreading and osteogenic differentiation of MSCs in the 2D culture, and the increased substrate stress relaxation promoted cell spreading and proliferation in the 2D culture and altered the cell morphology in the 3D culture [142]. In accordance with this, the promotion of cell spreading on various viscoelastic substrates has been reported in other cell types such as U2OS cells [140], myoblasts [143], and fibroblasts [142], in both 2D and 3D cell cultures. Moreover, substrate viscoelasticity also plays a regulatory role in cell migration, and substrates with faster stress relaxation promote the migration of cells such as myoblasts [143] and fibroblasts [142].

Both regulatory effects may be explained by focal adhesion (FA) formation and ligand clustering [128]. FA formation is probably the key mechanism through which the viscoelastic property of the substrate affects cell behaviors [140]. For instance, promoted FA formation was observed in hydrogels with faster relaxation (more viscoelastic). Chaudhuri et al. used hyaluronic acid and collagen I to form 3D hydrogels and found that the FA in MSCs was promoted by more viscoelastic hydrogels. The increased accumulation of β1 integrin, indicative of increased FA formation, was observed in the periphery of MSCs encapsulated in RGD-coupled ionically crosslinked alginate hydrogels with faster stress relaxation [142].

#### 5.2.2. Cell Proliferation

Viscoelastic matrices promote cell proliferation. Chaudhuri et al. showed that MSC proliferation was elevated in a PAM-alginate hydrogel with a faster relaxation rate [142]. Ryan et al. modified collagen hydrogels with insoluble elastin to induce prolonged stress relaxation (i.e., reduced viscosity), which resulted in lower proliferation and a more contractile phenotype of human smooth muscle cells (SMCs) [144]. Chao et al. seeded chondrocytes in chitosan-modified PLCL scaffolds with a viscoelastic property close to that of native bovine cartilage and observed that the cell proliferation was higher compared with that in unmodified (non-viscoelastic) scaffolds [145]. Peter et al. seeded preosteoblast cells (MC3T3-E1) on alginate-blended PCL scaffolds, and increased cell proliferation was found on viscoelastic scaffolds compared to pure PCL (low-viscoelasticity) scaffolds [42]. Finally, Tamate et al. showed that the proliferation of HeLa cells (cancer cells) was inhibited when the viscosity of the hydrogel was diminished [146]. The above studies all consistently demonstrated that substrate viscosity promotes cell proliferation in a variety of healthy and cancer cells.

#### 5.2.3. Cell Differentiation

The effect of substrate viscoelasticity on cell differentiation has been mostly studied in MSCs and the application of orthopedic tissue regeneration. For example, hydrogels with rapid stress relaxation induced the greater osteogenic differentiation of MSCs [147,148,149]. Viscoelastic hydrogels have also been successfully applied to regulate cell–cell and cell–matrix interactions for the differentiation and regeneration of bone and cartilage tissues with MSC spheroids [147,150]. The improved osteogenic differentiation of MSCs in faster relaxing (more viscoelastic) substrates has been related to mechanotransduction regulators such as the enhanced clustering of integrin ligands or stronger actomyosin contractility [142]. Li et al. prepared PAM hydrogels with different substrate stiffness to study cell proliferation. The substrate with slower stress relaxation drove the pro-inflammatory polarization of human bone-marrow-derived monocytes and their differentiation into antigen presenting cells, indicating an anti-inflammatory role of viscoelastic substrates [151].

#### 5.2.4. ECM Deposition

ECM deposition is a key outcome in the regeneration of connective tissues including bone and cartilage. Chondrocytes encapsulated in scaffolds with similar viscoelasticity to native cartilage tissue displayed the greater deposition of a cartilage-like matrix composed of type 2 collagen and aggrecan and the lower expression of type 1 collagen [152]. MSCs encapsulated in a viscoelastic hydrogel consisting of an interpenetrating network of alginate and fibrillar collagen type I with interferon-γ (IFN-γ)-loaded heparin-coated beads suppressed the proliferation of human T cells [153]. However, the results showed that cell proliferation was independent of substrate stiffness and was more dependent on the crosslinking components of the hydrogel.

### 5.3. Limitations of Current Viscoelastic Scaffolds

As an emerging area in tissue engineering and mechanobiology, the research into substrate viscoelasticity in cardiac applications is in its infancy stage. We summarized the reported viscoelastic properties of tissue-engineered scaffolds and native biological tissues in Table 3 and Table 4, respectively. Although the tissue-engineered scaffolds include a large range of viscosity (with the half relaxation time ranging from 10 s to 18,000 s), the elasticity is only at the low end (with a Young’s modulus <30 kPa and a storage modulus ranging from 0.04 kPa to 130 kPa). The elastic property is far below that of cardiac tissues (typically with a Young’s modulus of hundreds or thousands of kPa). Future studies should match both the elastic and viscous behavior of scaffolds to better replicate the physiological viscoelastic properties of cardiac tissues. In addition, the DMA technique (to obtain the storage and loss moduli) is seldom used for the measurement of cardiac tissues (Table 4). Different viscous parameters have been reported between the two research areas as well. While the half relaxation time is often provided for tissue-engineered scaffolds, the phase angle is more often obtained in native tissues. Therefore, it is difficult to compare the viscoelastic properties of tissue-engineered scaffolds to those of native cardiac tissues from the current literature. Future tissue engineering research should confirm the similarity of the viscoelastic properties of scaffolds and native tissues using measurements obtained via the same methodology.

**Figure 6:**
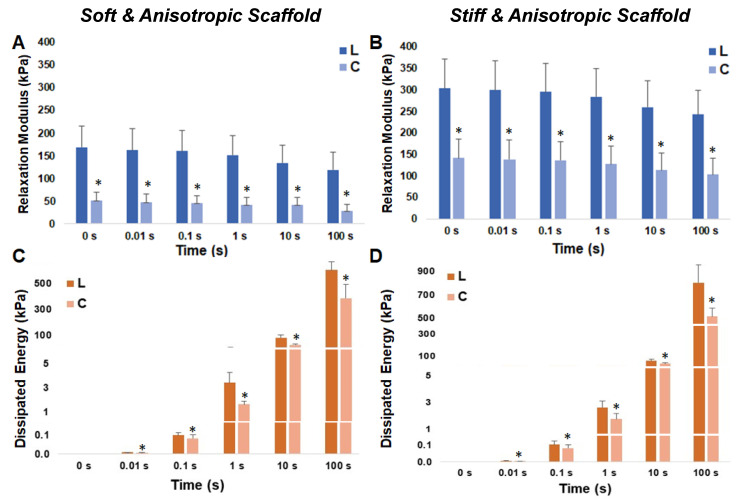
Viscoelastic properties of the previously reported anisotropic elastic scaffolds that mimic the stiffness of healthy (soft and anisotropic) and diseased (stiff and anisotropic) right ventricles [97]. Viscoelastic properties were measured by equibiaxial stress relaxation at the maximal strain of 15%. The elastic property was measured by the relaxation modulus (**A**,**B**), and the viscous property was measured by the dissipated energy (**C**,**D**), as described previously [90]. Results are shown as mean ± SE. The main fiber direction used was the longitudinal direction. * *p* < 0.05 between longitudinal (L) and circumferential (C) directions at the same relaxation time.

Furthermore, while the elastic property of cardiac bioscaffolds is often reported, it remains unknown whether they are viscoelastic. We recently reported different MSC responses to varied matrix stiffness and anisotropy degrees using PEUU scaffolds mimicking healthy and diseased right ventricles. The biaxial elastic behavior was measured in the main fiber and cross-fiber directions, and anisotropic elastic behavior was confirmed [97]. A re-examination of the two anisotropic scaffold groups that represent healthy (soft) and diseased (stiff) right ventricle elasticities was performed via stress relaxation tests. Unsurprisingly, viscoelastic behaviors were observed in these sheets. Moreover, we observed both elastic and viscous anisotropy in these scaffolds (Figure 6). Therefore, it is possible that the existing cardiac scaffolds present viscoelastic properties, although this behavior has been ignored.

## 6. Future Work

In summary, future work should focus on addressing the limitations of current scaffold fabrication techniques, such as the degree of anisotropy and the thickness limitation of hydrogel-based scaffolds. Additionally, efforts should be made to improve the repeatability and reproducibility of scaffold fabrication methods to ensure consistency across different studies or research groups and to allow for the easier comparison of results. Furthermore, there is a need for the appropriate characterization of scaffold mechanical properties and comparisons with the measurements obtained from myocardium tissues to ensure that engineered scaffolds exhibit the most important mechanical behaviors of native tissues. As biodegradation is expected in many tissue-engineered scaffolds, it is equally critical to investigate the mechanical changes in scaffolds during this process, data that are lacking in the current literature. Overall, continued efforts to improve scaffold design and fabrication techniques will enable the better investigation of the pathology of cardiac diseases and the development of patient-specific treatments for different types of HF, translating the research from bench to bedside.

## 7. Conclusions

Heart failure remains a major cause of morbidity and mortality worldwide, and tissue engineering offers promising therapeutic strategies for cardiac regeneration. The inclusion of biomimetic mechanical properties in cardiac scaffolds, such as anisotropy, nonlinear elasticity, and viscoelasticity, is crucial for promoting cell functions and myocardium tissue regeneration. This review summarized recent advances in cardiac scaffolds that achieved these mechanical properties, as well as the advantages and limitations of each method. The biological responses to tissue-specific mechanical environments were also discussed. In summary, this review highlighted the importance of considering mechanical properties in myocardium tissue engineering and regeneration. By developing biomimetic scaffolds, researchers and clinicians can create new opportunities to promote cardiac tissue regeneration and improve patient outcomes. These findings offer hope for the development of new therapeutic strategies to treat heart failure, the leading cause of death in the US and worldwide.

## Figures and Tables

**Table 3:** Viscoelastic properties of hydrogels reported in the literature. E refers to Young’s (elastic) modulus or initial modulus in stress relaxation. G′ is the storage modulus, and G″ is the loss modulus.

Material	Ligand	E (kPa)	G′ (kPa)	G″ (Pa)	Half Relaxation Time (τ_1/2_)	Ref.
Low and high MW alginate, alginate + PEG	RGD	9–17	N/A	N/A	Tens to thousands of seconds	[142]
Dynamic covalent crosslinked alginate	None	N/A	0.22–2.78 (at 1.6 Hz)	N/A	N/A	[135]
Agarose spheroids (ionically vs. covalently crosslinked)	Hydroxyapatite nanoparticles, BMP-2	N/A	2–6	0–0.75	20 s–infinity	[149]
PCL + alginate	None	N/A	0.0001–0.04 (at 0.1 Hz)	N/A	N/A	[42]
PDMS	None	N/A	3–130 (at 100 Hz)	1.5–3k (at 100 Hz)	N/A	[40]
GelMA or MeHA + decellularized ECM	Gelatin or hyaluronic acid	N/A	0.5–8 (at 1 Hz)	50–3000 (at 1 Hz)	N/A	[154]
Alginate hydrogel	RGD	9–17	3 (at 1 Hz)	0.1–0.8 (at 1 Hz)	60–3300 s	[142]
Polyacrylamide hydrogel	RGD	13.5	4.7 (at 1Hz)	N/A	N/A	[134]
Polyethylene glycol-based hydrogels	RGD	10	N/A	N/A	N/A	[143]
HA hydrogels	None	N/A	~5 (at 10 Hz)	~300 (at 10 Hz)	10–1000 s	[132]
Interpenetrating network of alginate and collagen I (NbTz)	Collagen	N/A	~1 (at 10 Hz)	~500 (at 10 Hz)	N/A	[133]
PAA	Collagen and fibronectin	N/A	~5.5 (at 0.16 Hz)	~10 (at 0.16 Hz)	N/A	[134]
Interpenetrating network of alginate and collagen I	Collagen I	0.009–0.51	N/A	N/A	55–18,000	[133]
Interpenetrating network of hyaluronic acid and collagen I	RGD	1.8–27	N/A	N/A	10–1000	[155]

**Table 4:** Viscoelastic properties of biological tissues reported in the literature. E refers to elastic/Young’s modulus or initial modulus in stress relaxation. G′ is the storage modulus, G″ is the loss modulus, and W_d_ is the dissipated energy. The phase angle was calculated as G″/G′.

Material	E (kPa)	G′ (kPa)	G″ (Pa)	Normalized Stress	W_d_ (kPa)	Damping	Dynamic Modulus (kPa)	Phase Angle (Radians)	Ref.
Bones	N/A	~90 (at 1 Hz)	~15 k (at 1 Hz)	N/A	N/A	N/A	N/A	~1.66 (at 1 Hz)	[156]
Cartilage	N/A	~45 k (at 5 Hz)	~9 k (at 5 Hz)	N/A	N/A	N/A	N/A	~0.2 (at 5 Hz)	[157]
Lungs	~1.4	N/A	~600	N/A	N/A	N/A	N/A	N/A	[158]
Brain	N/A	~3.5	~1 k	N/A	N/A	N/A	N/A	~0.28	[159]
Cornea	~19	~86	~12 k	N/A	N/A	N/A	N/A	~0.139	[160]
Liver	~30	~170	~17 k	N/A	N/A	N/A	N/A	~0.1	[161]
Right ventricle	~40–500	N/A	N/A	~0.706	~0.6	~0.3–0.7	N/A	N/A	[90]
Heart valve	N/A	~25 k (at 5 Hz)	~16 k (at 5 Hz)	N/A	N/A	N/A	N/A	~0.25	[162]
Pulmonary artery	N/A	N/A	N/A	N/A	N/A	~0.1–0.7 (at 0.01–10 Hz)	~60 (at 0.01 Hz)	N/A	[127]
Carotid artery	N/A	N/A	N/A	N/A	N/A	~0.2–0.3 (at 1 Hz)	N/A	N/A	[163]

## Data Availability

Data sharing not applicable.

## References

[1] Xu C., Okpokwasili C., Huang Y., Shi X., Wu J., Liao J., Tang L., Hong Y. (2020). Optimizing Anisotropic Polyurethane Scaffolds to Mechanically Match with Native Myocardium. ACS Biomater. Sci. Eng..

[2] Curtis M.W., Russell B. (2009). Cardiac Tissue Engineering. J. Cardiovasc. Nurs..

[3] Tsao C.W., Aday A.W., Almarzooq Z.I., Alonso A., Beaton A.Z., Bittencourt M.S., Boehme A.K., Buxton A.E., Carson A.P., Commodore-Mensah Y. (2022). Heart Disease and Stroke Statistics-2022 Update: A Report From the American Heart Association. Circulation.

[4] Boffito M., Sartori S., Ciardelli G. (2014). Polymeric scaffolds for cardiac tissue engineering: Requirements and fabrication technologies. Polym. Int..

[5] Nasr S.M., Rabiee N., Hajebi S., Ahmadi S., Fatahi Y., Hosseini M., Bagherzadeh M., Ghadiri A.M., Rabiee M., Jajarmi V. (2020). Biodegradable nanopolymers in cardiac tissue engineering: From concept towards nanomedicine. Int. J. Nanomed..

[6] Zhang M., Methot D., Poppa V., Fujio Y., Walsh K., Murry C.E. (2001). Cardiomyocyte Grafting for Cardiac Repair: Graft Cell Death and Anti-Death Strategies. J. Mol. Cell Cardiol..

[7] Kankala R.K., Zhu K., Sun X.N., Liu C.G., Wang S.B., Chen A.Z. (2018). Cardiac Tissue Engineering on the Nanoscale. ACS Biomater. Sci. Eng..

[8] Pomeroy J.E., Helfer A., Bursac N. (2020). Biomaterializing the promise of cardiac tissue engineering. Biotechnol. Adv..

[9] Huyer L.D., Zhang B., Korolj A., Montgomery M., Drecun S., Conant G., Zhao Y., Reis L., Radisic M. (2016). Highly Elastic and Moldable Polyester Biomaterial for Cardiac Tissue Engineering Applications. ACS Biomater. Sci. Eng..

[10] Janmey P.A., Miller R.T. (2011). Mechanisms of mechanical signaling in development and disease. J. Cell Sci..

[11] Engler A.J., Carag-Krieger C., Johnson C.P., Raab M., Tang H.-Y., Speicher D.W., Sanger J.W., Sanger J.M., Discher D.E. (2008). Embryonic cardiomyocytes beat best on a matrix with heart-like elasticity: Scar-like rigidity inhibits beating. J. Cell Sci..

[12] Dwyer K.D., Coulombe K.L.K. (2021). Cardiac mechanostructure: Using mechanics and anisotropy as inspiration for developing epicardial therapies in treating myocardial infarction. Bioact. Mater..

[13] Whitehead K.M., Hendricks H.K.L., Cakir S.N., de Castro Brás L.E. (2022). ECM roles and biomechanics in cardiac tissue decellularization. Am. J. Physiol.-Heart Circ. Physiol..

[14] Gilpin A., Yang Y. (2017). Decellularization Strategies for Regenerative Medicine: From Processing Techniques to Applications. Biomed Res. Int..

[15] Singelyn J.M., Sundaramurthy P., Johnson T.D., Schup-Magoffin P.J., Hu D.P., Faulk D.M., Wang J., Mayle K.M., Bartels K., Salvatore M. (2012). Catheter-deliverable hydrogel derived from decellularized ventricular extracellular matrix increases endogenous cardiomyocytes and preserves cardiac function post-myocardial infarction. J. Am. Coll. Cardiol..

[16] Fujita K., Tuchida Y., Seki H., Kosawada T., Feng Z., Shiraishi Y., Sato D., Nakamura T., Umezu M. Characterizing and modulating the mechanical properties of hydrogels from ventricular extracellular matrix. Proceedings of the 2015 10th Asian Control Conference (ASCC).

[17] Williams C., Quinn K.P., Georgakoudi I., Black L.D. (2014). Young developmental age cardiac extracellular matrix promotes the expansion of neonatal cardiomyocytes in vitro. Acta Biomater..

[18] Williams C., Sullivan K., Black L.D. (2015). Partially Digested Adult Cardiac Extracellular Matrix Promotes Cardiomyocyte Proliferation In Vitro. Adv. Healthc. Mater..

[19] Oberwallner B., Brodarac A., Anić P., Šarić T., Wassilew K., Neef K., Choi Y.-H., Stamm C. (2015). Human cardiac extracellular matrix supports myocardial lineage commitment of pluripotent stem cells ^†^. Eur. J. Cardio-Thorac. Surg..

[20] Johnson T.D., DeQuach J.A., Gaetani R., Ungerleider J., Elhag D., Nigam V., Behfar A., Christman K.L. (2014). Human versus porcine tissue sourcing for an injectable myocardial matrix hydrogel. Biomater. Sci..

[21] Johnson T.D., Hill R.C., Dzieciatkowska M., Nigam V., Behfar A., Christman K.L., Hansen K.C. (2016). Quantification of decellularized human myocardial matrix: A comparison of six patients. Proteom. Clin. Appl..

[22] Hirt M.N., Hansen A., Eschenhagen T. (2014). Cardiac Tissue Engineering. Circ. Res..

[23] Spang M.T., Christman K.L. (2018). Extracellular matrix hydrogel therapies: In vivo applications and development. Acta Biomater..

[24] Eschenhagen T., Fink C., Remmers U., Scholz H., Wattchow J., Weil J., Zimmermann W., Dohmen H.H., Schäfer H., Bishopric N. (1997). Three-dimensional reconstitution of embryonic cardiomyocytes in a collagen matrix: A new heart muscle model system. FASEB J..

[25] Cao D., Ding J. (2022). Recent advances in regenerative biomaterials. Regen. Biomater..

[26] Tsang V.L., Bhatia S.N. (2004). Three-dimensional tissue fabrication. Adv. Drug Deliv. Rev..

[27] Drury J.L., Mooney D.J. (2003). Hydrogels for tissue engineering: Scaffold design variables and applications. Biomaterials.

[28] Mantha S., Pillai S., Khayambashi P., Upadhyay A., Zhang Y., Tao O., Pham H.M., Tran S.D. (2019). Smart hydrogels in tissue engineering and regenerative medicine. Materials.

[29] Kesharwani P., Bisht A., Alexander A., Dave V., Sharma S. (2021). Biomedical applications of hydrogels in drug delivery system: An update. J. Drug Deliv. Sci. Technol..

[30] Hoare T.R., Kohane D.S. (2008). Hydrogels in drug delivery: Progress and challenges. Polymer.

[31] Ghasemiyeh P., Mohammadi-Samani S. (2019). Hydrogels as drug delivery systems; pros and cons. Trends Pharm. Sci..

[32] Wang C., Wang C., Huang Z., Xu S. (2018). Materials and structures toward soft electronics. Adv. Mater..

[33] Wirthl D., Pichler R., Drack M., Kettlguber G., Moser R., Gerstmayr R., Hartmann F., Bradt E., Kaltseis R., Siket C.M. (2017). Instant tough bonding of hydrogels for soft machines and electronics. Sci. Adv..

[34] Zhang A., Wang F., Chen L., Wei X., Xue M., Yang F., Jiang S. (2021). 3D printing hydrogels for actuators: A review. Chin. Chem. Lett..

[35] Ionov L. (2014). Hydrogel-based actuators: Possibilities and limitations. Mater. Today.

[36] Richter A. (2009). Hydrogels for actuators. Hydrogel Sensors and Actuators.

[37] Nguyen-Truong M., Li Y.V., Wang Z. (2020). Mechanical considerations of electrospun scaffolds for myocardial tissue and regenerative engineering. Bioengineering.

[38] Wu Y., Wang L., Guo B., Ma P.X. (2017). Interwoven Aligned Conductive Nanofiber Yarn/Hydrogel Composite Scaffolds for Engineered 3D Cardiac Anisotropy. ACS Nano.

[39] Ravishankar P., Ozkizilcik A., Husain A., Balachandran K. (2020). Anisotropic Fiber-Reinforced Glycosaminoglycan Hydrogels for Heart Valve Tissue Engineering. Tissue Eng. Part A.

[40] Petet T.J., Deal H.E., Zhao H.S., He A.Y., Tang C., Lemmon C.A. (2021). Rheological characterization of poly-dimethyl siloxane formulations with tunable viscoelastic properties. RSC Adv..

[41] Shahin-Shamsabadi A., Hashemi A., Tahriri M., Bastami F., Salehi M., Abbas F.M. (2018). Mechanical, material, and biological study of a PCL/bioactive glass bone scaffold: Importance of viscoelasticity. Mater. Sci. Eng. C.

[42] Kim Y.B., Kim G.H. (2015). PCL/Alginate Composite Scaffolds for Hard Tissue Engineering: Fabrication, Characterization, and Cellular Activities. ACS Comb. Sci..

[43] Formhals A. (1934). Process and Apparatus for Preparing Artificial Threads. U.S. Patent.

[44] Reneker D.H., Chun I. (1996). Nanometre diameter fibres of polymer, produced by electrospinning. Nanotechnology.

[45] Xue J., Wu T., Dai Y., Xia Y. (2019). Electrospinning and electrospun nanofibers: Methods, materials, and applications. Chem. Rev..

[46] Baghersad S., Hivechi A., Bahrami S.H., Milan P.B., Siegel R.A., Amoupour M. (2022). Optimal Aloe vera encapsulated PCL/Gel nanofiber design for skin substitute application and the evaluation of its in vivo implantation. J. Drug Deliv. Sci. Technol..

[47] Frenot A., Chronakis I.S. (2003). Polymer nanofibers assembled by electrospinning. Curr. Opin. Colloid. Interface Sci..

[48] Ayaz H.G.Ş., Perets A., Ayaz H., Gilroy K.D., Govindaraj M., Brookstein D., Lelkes P.I. (2014). Textile-templated electrospun anisotropic scaffolds for regenerative cardiac tissue engineering. Biomaterials.

[49] Madruga L.Y.C., Kipper M.J. (2022). Expanding the Repertoire of Electrospinning: New and Emerging Biopolymers, Techniques, and Applications. Adv. Healthc. Mater..

[50] Baghersad S., Bahrami S.H., Mohammadi M.R., Mojtahedi M.R.M., Milan P.B. (2018). Development of biodegradable electrospun gelatin/aloe-vera/poly (ε-caprolactone) hybrid nanofibrous scaffold for application as skin substitutes. Mater. Sci. Eng. C.

[51] Kanani A.G., Bahrami S.H. (2010). Review on electrospun nanofibers scaffold and biomedical applications. Trends Biomater. Artif. Organs.

[52] Bhardwaj N., Kundu S.C. (2010). Electrospinning: A fascinating fiber fabrication technique. Biotechnol. Adv..

[53] Kankala R.K., Zhu K., Li J., Wang C.-S., Wang S.-B., Chen A.-Z. (2017). Fabrication of arbitrary 3D components in cardiac surgery: From macro-, micro-to nanoscale. Biofabrication.

[54] Cornelissen D.-J., Faulkner-Jones A., Shu W. (2017). Current developments in 3D bioprinting for tissue engineering. Curr. Opin. Biomed. Eng..

[55] Noh S., Myung N., Park M., Kim S., Zhang S.-U., Kang H.-W., Kim B.W. (2018). 3D Bioprinting for Tissue Engineering. Clinical Regenerative Medicine in Urology.

[56] Xie Z., Gao M., Lobo A.O., Webster T.J. (2020). 3D bioprinting in tissue engineering for medical applications: The classic and the hybrid. Polymers.

[57] Wu S., Li Y., Zhang C., Tao L., Kuss M., Lim J.Y., Butcher J., Duan B. (2022). Tri-Layered and Gel-Like Nanofibrous Scaffolds with Anisotropic Features for Engineering Heart Valve Leaflets. Adv. Healthc. Mater..

[58] Hill M.R., Simon M.A., Valdez-Jasso D., Zhang W., Champion H.C., Sacks M.S. (2014). Structural and Mechanical Adaptations of Right Ventricle Free Wall Myocardium to Pressure Overload. Ann. Biomed. Eng..

[59] Avazmohammadi R., Hill M., Simon M., Sacks M. (2017). Transmural remodeling of right ventricular myocardium in response to pulmonary arterial hypertension. APL Bioeng..

[60] Datta P., Vyas V., Dhara S., Chowdhury A.R., Barui A. (2019). Anisotropy Properties of Tissues: A Basis for Fabrication of Biomimetic Anisotropic Scaffolds for Tissue Engineering. J. Bionic Eng..

[61] Tonndorf R., Aibibu D., Cherif C. (2021). Isotropic and anisotropic scaffolds for tissue engineering: Collagen, conventional, and textile fabrication technologies and properties. Int. J. Mol. Sci..

[62] Robinson A.J., Pérez-Nava A., Ali S.C., González-Campos J.B., Holloway J.L., Cosgriff-Hernandez E.M. (2021). Comparative analysis of fiber alignment methods in electrospinning. Matter.

[63] Korossis S. (2018). Structure-Function Relationship of Heart Valves in Health and Disease.

[64] Adadi N., Yadid M., Gal I., Asulin M., Feiner R., Edri R., Dvir T. (2020). Electrospun Fibrous PVDF-TrFe Scaffolds for Cardiac Tissue Engineering, Differentiation, and Maturation. Adv. Mater. Technol..

[65] Markatos D.N., Sarakinis A., Mavrilas D. (2018). Tuning Fiber Alignment to Achieve Mechanical Anisotropy on Polymeric Electrospun Scaffolds for Cardiovascular Tissue Engineering. J. Mater. Sci. Eng..

[66] Long Y.-Z., Yu M., Sun B., Gu C.-Z., Fan Z. (2012). Recent advances in large-scale assembly of semiconducting inorganic nanowires and nanofibers for electronics, sensors and photovoltaics. Chem. Soc. Rev..

[67] Courtney T., Sacks M.S., Stankus J., Guan J., Wagner W.R. (2006). Design and analysis of tissue engineering scaffolds that mimic soft tissue mechanical anisotropy. Biomaterials.

[68] Liu W., Nguyen-Truong M., LeBar K., Labus K.M., Gray E., Ahern M., Neelakantan S., Avazmohammadi R., McGilvray K.C., Puttlitz C.M. (2022). Multiscale Contrasts Between the Right and Left Ventricle Biomechanics in Healthy Adult Sheep and Translational Implications. Front. Bioeng. Biotechnol..

[69] Yee W.A., Nguyen A.C., Lee P.S., Kotaki M., Liu Y., Tan B.T., Mhaisalkar S., Lu X. (2008). Stress-induced structural changes in electrospun polyvinylidene difluoride nanofibers collected using a modified rotating disk. Polym. Guildf.

[70] Park S.A., Park K., Yoon H., Son J.G., Min T., Kim G.H. (2007). Apparatus for preparing electrospun nanofibers: Designing an electrospinning process for nanofiber fabrication. Polym. Int..

[71] Zhao G., Feng Y., Xue L., Cui M., Zhang Q., Xu F., Peng N., Jiang Z., Gao D., Zhang X. (2022). Anisotropic conductive reduced graphene oxide/silk matrices promote post-infarction myocardial function by restoring electrical integrity. Acta Biomater..

[72] McClure M.J., Sell S.A., Ayres C.E., Simpson D.G., Bowlin G.L. (2009). Electrospinning-aligned and random polydioxanone–polycaprolactone–silk fibroin-blended scaffolds: Geometry for a vascular matrix. Biomed. Mater..

[73] Teo W.E., Ramakrishna S. (2006). A review on electrospinning design and nanofibre assemblies. Nanotechnology.

[74] Hobson C.M., Amoroso N.J., Amini R., Ungchusri E., Hong Y., D’Amore A., Sacks M.S., Wagner W.R. (2015). Fabrication of elastomeric scaffolds with curvilinear fibrous structures for heart valve leaflet engineering. J. Biomed. Mater. Res. A.

[75] Xie J., MacEwan M.R., Schwartz A.G., Xia Y. (2010). Electrospun nanofibers for neural tissue engineering. Nanoscale.

[76] Jha B.S., Colello R.J., Bowman J.R., Sell S.A., Lee K.D., Bigbee J.W., Bowlin G.L., Chow W.N., Mathern B.E., Simpson D.G. (2011). Two pole air gap electrospinning: Fabrication of highly aligned, three-dimensional scaffolds for nerve reconstruction. Acta Biomater..

[77] Linder H.R., Glass A.A., Day D.E., Sell S.A. (2020). Manipulating air-gap electrospinning to create aligned polymer nanofiber-wrapped glass microfibers for cortical bone tissue engineering. Bioengineering.

[78] Lei T., Xu Z., Cai X., Xu L., Sun D. (2018). New Insight into Gap Electrospinning: Toward Meter-long Aligned Nanofibers. Langmuir.

[79] Cai X., Zhu P., Lu X., Liu Y., Lei T., Sun D. (2017). Electrospinning of very long and highly aligned fibers. J. Mater. Sci..

[80] Mao M., He J., Li Z., Han K., Li D. (2020). Multi-directional cellular alignment in 3D guided by electrohydrodynamically-printed microlattices. Acta Biomater..

[81] Lei Q., He J., Li D. (2019). Electrohydrodynamic 3D printing of layer-specifically oriented, multiscale conductive scaffolds for cardiac tissue engineering. Nanoscale.

[82] Kim W., Kim M., Kim G.H. (2018). 3D-Printed Biomimetic Scaffold Simulating Microfibril Muscle Structure. Adv. Funct. Mater..

[83] Kim W., Jang C.H., Kim G.H. (2019). A Myoblast-Laden Collagen Bioink with Fully Aligned Au Nanowires for Muscle-Tissue Regeneration. Nano Lett..

[84] Cui H., Liu C., Esworthy T., Huang Y., Yu Z., Zhou X., San H., Lee S., Hann S.Y., Boehm M. (2022). 4D physiologically adaptable cardiac patch: A 4-month in vivo study for the treatment of myocardial infarction. Sci. Adv..

[85] Kato B., Wisser G., Agrawal D.K., Wood T., Thankam F.G. (2021). 3D bioprinting of cardiac tissue: Current challenges and perspectives. J. Mater. Sci. Mater. Med..

[86] Wang Z., Wang L., Li T., Liu S., Guo B., Huang W., Wu Y. (2021). 3D bioprinting in cardiac tissue engineering. Theranostics.

[87] Xing J., Liu N., Xu N., Chen W., Xing D. (2022). Engineering Complex Anisotropic Scaffolds beyond Simply Uniaxial Alignment for Tissue Engineering. Adv. Funct. Mater..

[88] Ren M., Ong C.W., Buist M.L., Yap C.H. (2022). Biventricular biaxial mechanical testing and constitutive modelling of fetal porcine myocardium passive stiffness. J. Mech. Behav. Biomed. Mater..

[89] Sommer G., Schriefl A.J., Andrä M., Sacherer M., Viertler C., Wolinski H., Holzapfel G.A. (2015). Biomechanical properties and microstructure of human ventricular myocardium. Acta Biomater..

[90] Liu W., Nguyen-Truong M., Ahern M., Labus K.M., Puttlitz C.M., Wang Z. (2021). Different Passive Viscoelastic Properties Between the Left and Right Ventricles in Healthy Adult Ovine. J. Biomech. Eng..

[91] Jang S., Vanderpool R.R., Avazmohammadi R., Lapshin E., Bachman T.N., Sacks M., Simon M.A. (2023). Biomechanical and Hemodynamic Measures of Right Ventricular Diastolic Function: Translating Tissue Biomechanics to Clinical Relevance. J. Am. Heart Assoc..

[92] Liu W., Nguyen-Truong M., Labus K., Boon J., Easley J., Monnet E., Puttlitz C., Wang Z. (2020). Correlations between the right ventricular passive elasticity and organ function in adult ovine. J. Integr. Cardiol..

[93] Nicosia M.A., Kasalko J.S., Cochran R.P., Einstein D.R., Kunzelman K.S. (2002). Biaxial mechanical properties of porcine ascending aortic wall tissue. J. Heart Valve Dis..

[94] Billiar K.L., Sacks M.S. (1999). Biaxial Mechanical Properties of the Natural and Glutaraldehyde Treated Aortic Valve Cusp—Part I: Experimental Results. J. Biomech. Eng..

[95] Balguid A., Rubbens M.P., Mol A., Bank R.A., Bogers A.J.J.C., van Kats J.P., de Mol B.A.J.M., Baaijens F.P.T., Bouten C.V.C. (2007). The role of collagen cross-links in biomechanical behavior of human aortic heart valve leaflets—Relevance for tissue engineering. Tissue Eng..

[96] Kai D., Prabhakaran M.P., Jin G., Ramakrishna S. (2011). Guided orientation of cardiomyocytes on electrospun aligned nanofibers for cardiac tissue engineering. J. Biomed. Mater. Res. B Appl. Biomater..

[97] Nguyen-Truong M., Kim S., Doherty C., Frederes M., LeBar K., Ghosh S., Hematti P., Chinnadurai R., Wagner W.R., Wang Z. (2022). Pro-angiogenic Potential of Mesenchymal Stromal Cells Regulated by Matrix Stiffness and Anisotropy Mimicking Right Ventricles. Biomacromolecules.

[98] Stankus J.J., Guan J., Fujimoto K., Wagner W.R. (2006). Microintegrating smooth muscle cells into a biodegradable, elastomeric fiber matrix. Biomaterials.

[99] Suhaeri M., Subbiah R., Kim S.-H., Kim C.-H., Oh S.J., Kim S.-H., Park K. (2017). Novel Platform of Cardiomyocyte Culture and Coculture via Fibroblast-Derived Matrix-Coupled Aligned Electrospun Nanofiber. ACS Appl. Mater. Interfaces.

[100] Reid J.A., Dwyer K.D., Schmitt P.R., Soepriatna A.H., Coulombe K.L.K., Callanan A. (2021). Architected fibrous scaffolds for engineering anisotropic tissues. Biofabrication.

[101] Eom S., Park S.M., Hwang D.G., Kim H.W., Jang J., Kim D.S. (2021). Fabrication of an align-random distinct, heterogeneous nanofiber mat endowed with bifunctional properties for engineered 3D cardiac anisotropy. Compos. B Eng..

[102] Jahnavi S., Saravanan U., Arthi N., Bhuvaneshwar G.S., Kumary T.V., Rajan S., Verma R.S. (2017). Biological and mechanical evaluation of a Bio-Hybrid scaffold for autologous valve tissue engineering. Mater. Sci. Eng. C.

[103] Fomovsky G.M., Macadangdang J.R., Ailawadi G., Holmes J.W. (2011). Model-Based Design of Mechanical Therapies for Myocardial Infarction. J. Cardiovasc. Transl. Res..

[104] Fomovsky G.M., Clark S.A., Parker K.M., Ailawadi G., Holmes J.W. (2012). Anisotropic Reinforcement of Acute Anteroapical Infarcts Improves Pump Function. Circ. Heart Fail..

[105] Sallin E.A. (1969). Fiber Orientation and Ejection Fraction in the Human Left Ventricle. Biophys. J..

[106] Sefton M.V., Simmons C.A. (2022). Hearts by design. Science.

[107] Chang H., Liu Q., Zimmerman J.F., Lee K.Y., Jin Q., Peters M.M., Rosnach M., Choi S., Kim S.L., Ardoña H.A.M. (2022). Recreating the heart’s helical structure-function relationship with focused rotary jet spinning. Science.

[108] Camman M., Joanne P., Agbulut O., Hélary C. (2022). 3D models of dilated cardiomyopathy: Shaping the chemical, physical and topographical properties of biomaterials to mimic the cardiac extracellular matrix. Bioact. Mater..

[109] Wanjare M., Hou L., Nakayama K.H., Kim J.J., Mezak N.P., Abilez O.J., Tzatzalos E., Wu J.C., Huang N.F. (2017). Anisotropic microfibrous scaffolds enhance the organization and function of cardiomyocytes derived from induced pluripotent stem cells. Biomater. Sci..

[110] Abbasi A., Imaichi S., Ling V., Shukla A. (2022). Mesenchymal Stem Cell Behavior on Soft Hydrogels with Aligned Surface Topographies. ACS Appl. Bio Mater..

[111] Islam A., Younesi M., Mbimba T., Akkus O. (2016). Collagen substrate stiffness anisotropy affects cellular elongation, nuclear shape, and stem cell fate toward anisotropic tissue lineage. Adv. Healthc. Mater..

[112] Zhang W., Wang Z., Xie C., Wang X., Luo F., Hong M., Zhou R., Ma C., Lin N., Zhang J. (2019). Scaffold with micro/macro-architecture for myocardial alignment engineering into complex 3D cell patterns. Adv. Healthc. Mater..

[113] Guan J., Wang F., Li Z., Chen J., Guo X., Liao J., Moldovan N.I. (2011). The stimulation of the cardiac differentiation of mesenchymal stem cells in tissue constructs that mimic myocardium structure and biomechanics. Biomaterials.

[114] Kadir N.D., Yang Z., Hassan A., Denslin V., Lee E.H. (2021). Electrospun fibers enhanced the paracrine signaling of mesenchymal stem cells for cartilage regeneration. Stem Cell Res. Ther..

[115] Baker B.M., Nathan A.S., Gee A.O., Mauck R.L. (2010). The influence of an aligned nanofibrous topography on human mesenchymal stem cell fibrochondrogenesis. Biomaterials.

[116] Delaine-Smith R.M., Hann A.J., Green N.H., Reilly G.C. (2021). Electrospun Fiber Alignment Guides Osteogenesis and Matrix Organization Differentially in Two Different Osteogenic Cell Types. Front. Bioeng. Biotechnol..

[117] Allen A.C.B., Barone E., Momtahan N., Crosby C.O., Tu C., Deng W., Polansky K., Zoldan J. (2019). Temporal Impact of Substrate Anisotropy on Differentiating Cardiomyocyte Alignment and Functionality. Tissue Eng. Part A.

[118] Vélez-Rendón D., Pursell E.R., Shieh J., Valdez-Jasso D. (2019). Relative Contributions of Matrix and Myocytes to Biaxial Mechanics of the Right Ventricle in Pulmonary Arterial Hypertension. J. Biomech. Eng..

[119] Meng X., Wang X., Zhang B., Zhang Y., Jiang Y., Guo M., Li Q. (2021). Fibrous scaffold with a tunable nonlinear elasticity. Polym. Test..

[120] Niu Z., Wang X., Meng X., Guo X., Jiang Y., Xu Y., Li Q., Shen C. (2019). Controllable fiber orientation and nonlinear elasticity of electrospun nanofibrous small diameter tubular scaffolds for vascular tissue engineering. Biomed. Mater..

[121] Szczesny S.E., Driscoll T.P., Tseng H.-Y., Liu P.-C., Heo S.-J., Mauck R.L., Chao P.-H.G. (2017). Crimped Nanofibrous Biomaterials Mimic Microstructure and Mechanics of Native Tissue and Alter Strain Transfer to Cells. ACS Biomater. Sci. Eng..

[122] Zhang Y., Wang X., Li K., Zhang Y., Yu X., Wang H., Wu X., Shi Z., Liu L., Zheng W. (2022). Nanofibrous tissue engineering scaffold with nonlinear elasticity created by controlled curvature and porosity. J. Mech. Behav. Biomed. Mater..

[123] Ramaraju H., Ul-Haque A., Verga A.S., Bocks M.L., Hollister S.J. (2020). Modulating nonlinear elastic behavior of biodegradable shape memory elastomer and small intestinal submucosa(SIS) composites for soft tissue repair. J. Mech. Behav. Biomed. Mater..

[124] Hall M.S., Alisafaei F., Ban E., Feng X., Hui C.-Y., Shenoy V.B., Wu M. (2016). Fibrous nonlinear elasticity enables positive mechanical feedback between cells and ECMs. Proc. Natl. Acad. Sci. USA.

[125] Liu K., Mihaila S.M., Rowan A., Oosterwijk E., Kouwer P.H.J. (2019). Synthetic Extracellular Matrices with Nonlinear Elasticity Regulate Cellular Organization. Biomacromolecules.

[126] Surrao D.C., Fan J.C.Y., Waldman S.D., Amsden B.G. (2012). A crimp-like microarchitecture improves tissue production in fibrous ligament scaffolds in response to mechanical stimuli. Acta Biomater..

[127] Wang Z., Lakes R.S., Golob M., Eickhoff J.C., Chesler N.C. (2013). Changes in Large Pulmonary Arterial Viscoelasticity in Chronic Pulmonary Hypertension. PLoS ONE.

[128] Huang D., Huang Y., Xiao Y., Yang X., Lin H., Feng G., Zhu X., Zhang X. (2019). Viscoelasticity in natural tissues and engineered scaffolds for tissue reconstruction. Acta Biomater..

[129] Li R.L., Russ J., Paschalides C., Ferrari G., Waisman H., Kysar J.W., Kalfa D. (2019). Mechanical considerations for polymeric heart valve development: Biomechanics, materials, design and manufacturing. Biomaterials.

[130] Dokos S., Smaill B.H., Young A.A., LeGrice I.J. (2002). Shear properties of passive ventricular myocardium. Am. J. Physiol. Heart Circ. Physiol..

[131] Abidine Y., Giannetti A., Revilloud J., Laurent V.M., Verdier C. (2021). Viscoelastic Properties in Cancer: From Cells to Spheroids. Cells.

[132] Ma Y., Han T., Yang Q., Wang J., Feng B., Jia Y., Wei Z., Xu F. (2021). Viscoelastic Cell Microenvironment: Hydrogel-Based Strategy for Recapitulating Dynamic ECM Mechanics. Adv. Funct. Mater..

[133] Vining K.H., Stafford A., Mooney D.J. (2019). Sequential modes of crosslinking tune viscoelasticity of cell-instructive hydrogels. Biomaterials.

[134] Charrier E.E., Pogoda K., Wells R.G., Janmey P.A. (2018). Control of cell morphology and differentiation by substrates with independently tunable elasticity and viscous dissipation. Nat. Commun..

[135] Morgan F.L.C., Fernández-Pérez J., Moroni L., Baker M.B. (2022). Tuning Hydrogels by Mixing Dynamic Cross-Linkers: Enabling Cell-Instructive Hydrogels and Advanced Bioinks. Adv. Healthc. Mater..

[136] Richardson B.M., Walker C.J., Macdougall L.J., Hoye J.W., Randolph M.A., Bryant S.J., Anseth K.S. (2020). Viscoelasticity of hydrazone crosslinked poly(ethylene glycol) hydrogels directs chondrocyte morphology during mechanical deformation. Biomater. Sci..

[137] Pauly H.M., Place L.W., Donahue T.L.H., Kipper M.J. (2017). Mechanical Properties and Cell Compatibility of Agarose Hydrogels Containing Proteoglycan Mimetic Graft Copolymers. Biomacromolecules.

[138] Mondésert H., Bossard F., Favier D. (2021). Anisotropic electrospun honeycomb polycaprolactone scaffolds: Elaboration, morphological and mechanical properties. J. Mech. Behav. Biomed. Mater..

[139] Cameron A.R., Frith J.E., Cooper-White J.J. (2011). The influence of substrate creep on mesenchymal stem cell behaviour and phenotype. Biomaterials.

[140] Chaudhuri O., Gu L., Darnell M., Klumpers D., Bencherif S.A., Weaver J.C., Huebsch N., Mooney D.J. (2015). Substrate stress relaxation regulates cell spreading. Nat. Commun..

[141] Shafiq M., Ali O., Han S.-B., Kim D.-H. (2021). Mechanobiological Strategies to Enhance Stem Cell Functionality for Regenerative Medicine and Tissue Engineering. Front. Cell Dev. Biol..

[142] Chaudhuri O., Gu L., Klumpers D., Darnell M., Bencherif S.A., Weaver J.C., Huebsch N., Lee H., Lippens E., Duda G.N. (2016). Hydrogels with tunable stress relaxation regulate stem cell fate and activity. Nat. Mater..

[143] McKinnon D.D., Domaille D.W., Cha J.N., Anseth K.S. (2014). Biophysically Defined and Cytocompatible Covalently Adaptable Networks as Viscoelastic 3D Cell Culture Systems. Adv. Mater..

[144] Ryan A.J., O’Brien F.J. (2015). Insoluble elastin reduces collagen scaffold stiffness, improves viscoelastic properties, and induces a contractile phenotype in smooth muscle cells. Biomaterials.

[145] Li C., Wang L., Yang Z., Kim G., Chen H., Ge Z. (2012). A Viscoelastic Chitosan-Modified Three-Dimensional Porous Poly(L-Lactide-co-ε-Caprolactone) Scaffold for Cartilage Tissue Engineering. J. Biomater. Sci. Polym. Ed..

[146] Tamate R., Ueki T., Kitazawa Y., Kuzunuki M., Watanabe M., Akimoto A.M., Yoshida R. (2016). Photo-Dimerization Induced Dynamic Viscoelastic Changes in ABA Triblock Copolymer-Based Hydrogels for 3D Cell Culture. Chem. Mater..

[147] Wu D.T., Jeffreys N., Diba M., Mooney D.J. (2022). Viscoelastic Biomaterials for Tissue Regeneration. Tissue Eng. Part C Methods.

[148] Chaudhuri O., Cooper-White J., Janmey P.A., Mooney D.J., Shenoy V.B. (2020). Effects of extracellular matrix viscoelasticity on cellular behavior. Nature.

[149] Whitehead J., Griffin K.H., Gionet-Gonzales M., Vorwald C.E., Cinque S.E., Leach J.K. (2021). Hydrogel mechanics are a key driver of bone formation by mesenchymal stromal cell spheroids. Biomaterials.

[150] Ho S.S., Keown A.T., Addison B., Leach J.K. (2017). Cell Migration and Bone Formation from Mesenchymal Stem Cell Spheroids in Alginate Hydrogels Are Regulated by Adhesive Ligand Density. Biomacromolecules.

[151] Li X., Sun Q., Li Q., Kawazoe N., Chen G. (2018). Functional Hydrogels with Tunable Structures and Properties for Tissue Engineering Applications. Front. Chem..

[152] Irawan V., Sung T.-C., Higuchi A., Ikoma T. (2018). Collagen Scaffolds in Cartilage Tissue Engineering and Relevant Approaches for Future Development. Tissue Eng. Regen. Med..

[153] Gonzalez-Pujana A., Vining K.H., Zhang D.K.Y., Santos-Vizcaino E., Igartua M., Hernandez R.M., Mooney D.J. (2020). Multifunctional biomimetic hydrogel systems to boost the immunomodulatory potential of mesenchymal stromal cells. Biomaterials.

[154] Basara G., Ozcebe S.G., Ellis B.W., Zorlutuna P. (2021). Tunable Human Myocardium Derived Decellularized Extracellular Matrix for 3D Bioprinting and Cardiac Tissue Engineering. Gels.

[155] Lou J., Stowers R., Nam S., Xia Y., Chaudhuri O. (2018). Stress relaxing hyaluronic acid-collagen hydrogels promote cell spreading, fiber remodeling, and focal adhesion formation in 3D cell culture. Biomaterials.

[156] Liao H.-T., Chen C.-T., Chen J.-P. (2011). Osteogenic Differentiation and Ectopic Bone Formation of Canine Bone Marrow-Derived Mesenchymal Stem Cells in Injectable Thermo-Responsive Polymer Hydrogel. Tissue Eng. Part C Methods.

[157] Lawless B.M., Sadeghi H., Temple D.K., Dhaliwal H., Espino D.M., Hukins D.W.L. (2017). Viscoelasticity of articular cartilage: Analysing the effect of induced stress and the restraint of bone in a dynamic environment. J. Mech. Behav. Biomed. Mater..

[158] Jorba I., Beltrán G., Falcones B., Suki B., Farré R., García-Aznar J.M., Navajas D. (2019). Nonlinear elasticity of the lung extracellular microenvironment is regulated by macroscale tissue strain. Acta Biomater..

[159] Ozkaya E., Fabris G., Macruz F., Suar Z.M., Abderezaei J., Su B., Laksari K., Wu L., Camarillo D.B., Pauly K.B. (2021). Viscoelasticity of children and adolescent brains through MR elastography. J. Mech. Behav. Biomed. Mater..

[160] Kazaili A., Geraghty B., Akhtar R. (2019). Microscale assessment of corneal viscoelastic properties under physiological pressures. J. Mech. Behav. Biomed. Mater..

[161] Estermann S.J., Pahr D.H., Reisinger A. (2020). Hyperelastic and viscoelastic characterization of hepatic tissue under uniaxial tension in time and frequency domain. J. Mech. Behav. Biomed. Mater..

[162] Alhadrami H.A., Syed R.U.R., Zahid A.A., Ahmed R., Hasan S., Hasan A. (2019). Structure and Rheological Properties of Bovine Aortic Heart Valve and Pericardium Tissue: Implications in Bioprosthetic and Tissue-Engineered Heart Valves. J. Healthc. Eng..

[163] Tian L., Wang Z., Lakes R.S., Chesler N.C. (2013). Comparison of Approaches to Quantify Arterial Damping Capacity from Pressurization Tests on Mouse Conduit Arteries. J. Biomech. Eng..

